# Suppression of bone remodeling associated with long-term bisphosphonate treatment is mediated by microRNA-30a-5p

**DOI:** 10.1080/21655979.2022.2060584

**Published:** 2022-04-12

**Authors:** Xia Li, Ruisheng Xu, Jun-Xing Ye, Feng-Lai Yuan

**Affiliations:** a Institute of Integrated Chinese and Western Medicine, The Hospital Affiliated to Jiangnan University, Wuxi, Jiangsu, China; b Department of Orthopaedics and Central Laboratory, The Third Hospital Affiliated to Nantong University, Wuxi, Jiangsu, China; c Department of Orthopaedics, The Hospital Affiliated to Jiangnan University, Wuxi, Jiangsu, China

**Keywords:** Osteoporosis, bisphosphonate, MiRNA, bone loss, osteoblast

## Abstract

Oral bisphosphonates (BPs) are a first-line treatment for osteoporosis. It is becoming a hot topic to identify new indicators for the early prediction of therapeutic effects and adverse reactions during the long-term use of BPs. To determine whether microRNA (miRNA) expression is modulated by long-term BPs treatment, we performed miRNA expression profiling analysis in patients receiving long-term BP treatment for postmenopausal OP. To assess the effect of BPs on miRNA expression, we used an Affymetrix Genechip miRNA array to analyze serum samples obtained from postmenopausal OP patients on long-term BP treatment and healthy controls. MiRNAs affected by BPs and their predicted targets were examined. We also investigated the effects of miRNA on osteoblast differentiation in vitro and on ovariectomy-induced bone loss in vivo. We observed that the level of miR-30a-5p was significantly increased in patients receiving long-term BP treatment for postmenopausal OP. Furthermore, miR-30a-5p was negatively correlated with bone formation. Consistent with this, in vitro osteoblast activity and matrix mineralization were increased by an antagomir of miR-30a-5p and decreased by an agomir of miR-30a-5p. We also found that miR-30a-5p directly targeted RUNX1 to inhibit osteoblastic differentiation. Consistent with the in vitro results, miR-30a-5p antagomir administration promoted bone formation in ovariectomized mice. Our findings identified miR-30a-5p as a novel mediator of long-term BP treatment that regulates bone formation in postmenopausal OP patients.

## GA

**Figure:**
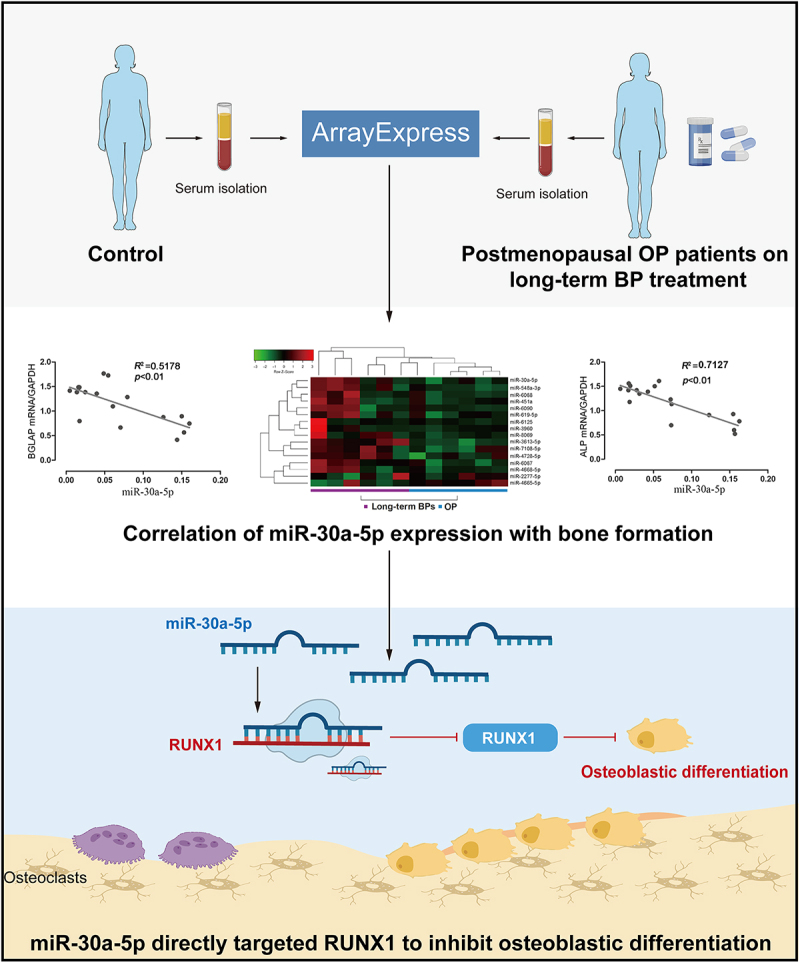

## 1. Introduction

Osteoporosis (OP) is a condition characterized by low bone mass and increased bone fragility, putting patients at risk of fractures [1]. Indeed, it is estimated that every 3 s, an osteoporotic fracture occurs around the world. Vertebral and hip fractures are a hallmark of OP, and are common among elderly people and postmenopausal women. Unfortunately, osteoporotic fractures are associated with high morbidity and mortality, and most patients with such fractures become unable to perform the activities of daily living [2]. Currently, OP imposes a massive burden on the lives and work of individuals and the society, and strategies for its effective prevention, diagnosis, and treatment are urgently needed.

Advanced age, genetics, low calcium and vitamin D intake, thinness, and menopause are the main risk factors for OP [3]. Postmenopausal OP, which is a major subtype of OP, is caused by endogenous estrogen deficiency in postmenopausal women [4]. Estrogen inhibits the differentiation and survival of osteoclasts while promoting the survival of osteoblasts; therefore, the bone loss caused by estrogen deficiency is at least partially explained by increased bone resorption by osteoclasts [5,6].

As OP is a chronic disease, it is conceivable that the treatment for OP can last for the rest of the patient’s life. Current drug treatments for postmenopausal OP aim to prevent fractures by inhibiting bone resorption and stimulating bone formation. Oral bisphosphonates (BPs) are a first-line treatment for postmenopausal OP and are well effective in the prevention of fragility fractures; these drugs decrease bone turnover by inhibiting osteoclast function [7]. However, oral BPs produce only a moderate increase in bone mineral density and have limited effects on the risk factors for fracture [8]. Additionally, the long-term use of BPs is associated with a risk of severe upper gastrointestinal complications [9]. Moreover, all BPs are contraindicated in patients with severe renal impairment, as the intravenous administration of BPs, especially at high dosage, has been linked to acute renal failure in patients with malignancy [10]. Furthermore, there is much evidence linking the long-term use of BPs with suppression of bone remodeling, which leads to an increased risk of atypical femur fractures [11]. BP-induced suppression of bone remodeling is attributed to the inhibition of osteoclast activity, and causes ‘frozen bone,’ which reduces the quality of bone and can induce skeletal fragility and osteonecrosis of the jaw [12]. Unfortunately, there is a lack of indicators for the early prediction of therapeutic effects and adverse reactions during the long-term use of BPs for the treatment of OP patients [13]. Moreover, the mechanism underlying BP-induced skeletal fragility is unknown; an understanding of this process may lead to the discovery of novel biomarkers for the management of postmenopausal OP with long-term BP treatment.

MicroRNAs (miRNAs) are small non-coding mRNA transcripts that can degrade or inhibit mRNA transcripts, and thereby inhibit protein levels [14]. Studies have shown that miRNAs are essential for the regulation of osteogenesis and osteoclastogenesis [15,16]. Since their discovery, miRNAs and their targets have been identified as novel regulators of bone-related pathologies, such as OP [17]. As miRNAs are actively released from bone cells into biological fluids, they are regarded as important biomarkers of bone-related diseases [18]. To date, circulating miRNAs have been associated with many diseases. Indeed, miRNAs have been successfully annotated to specific biological functions and diseases, and are now regarded as ‘fingerprints’ for specific diseases. Circulating miRNA signatures have become a novel class of biomarkers and even potential targets for future therapies, and are currently being investigated as biomarkers for the identification of molecular alterations that are distinctive for OP [19].

In the present study, we hypothesized that long-term BP treatment for postmenopausal OP was associated with an altered miRNA profile. To validate this hypothesis, we analyzed the alterations in miRNA expression associated with long-term BP treatment in postmenopausal OP patients. We then evaluated whether the altered miRNAs identified in the OP patients inhibited osteoblastic differentiation in MC3T3-E1 cells, an osteoblastic cell line, and whether they regulated bone formation in a murine OP model.

## 2. Materials and methods

### 2.1. Study design and patient selection

This study involved 65 postmenopausal women (mean age, 66.9 ± 6.6 years; range, 58–86 years) with OP who visited the outpatient clinic of the Hospital Affiliated to Jiangnan University, Wuxi, Jiangsu, China. Patients who were treated with oral BPs (70 mg/wk, n = 30) for 1–5 years and received vitamin D (800 IU/d) and calcium (1200 mg/d) supplementation were included in the BP group. OP patients with or without atypical fractures were included. Patients with OP who were treated without oral BPs were included as a control group (n = 30). Patients with a history of any of the following in the previous 5 years were excluded from this study: malignancy, coronary heart disease, diabetes, uncontrolled thyroid disease, or any other severe disease. We measured the bone mineral density (BMD; in g/cm^2^) of the total hip and the lumbar spine (L1–L4) by using a GE-Lunar DPX-NT dual-energy X-ray absorptiometry device (GE Healthcare, Madison, WI, USA). Ethical approval for the study protocol was obtained from the institutional review board of The Hospital Affiliated to Jiangnan University, and all participants provided informed consent. Peripheral whole blood samples were collected from all participants. Serum specimens were extracted within 1 h of sample collection, and stored in 5-mL aliquots in plastic tubes at −80°C for the analysis of miRNA, bone gamma carboxyglutamate protein (BGLAP), and alkaline phosphatase (ALP) expressions.

### 2.2. RNA isolation

Total small RNAs were extracted from the serum and bone specimens by using TRIzol and assessed on a NanoDrop 2000 spectrophotometer (Thermo Fisher Scientific, Waltham, MA, USA) and an Agilent 2100 Bioanalyzer (Agilent Technologies, Palo Alto, CA, USA). The purity of the extracted RNAs was ascertained using A260/A280 ratios of 1.7–2.2, as measured on the Agilent 2100 Bioanalyzer, and only RNAs with sufficient purity were used for the subsequent analyses. All RNA samples were stored at −80°C until reverse transcription and quantitative real-time polymerase chain reaction (qRT-PCR).

### 2.3. Determination of miRNA expression profiles

The protocol for the microarray experiments has been previously described [20]. In brief, total RNAs were amplified using a GeneChip miRNA 4.0 Expression Kit (Affymetrix, Santa Clara, CA, USA). The amplified RNAs were labeled, subjected to microarray hybridization, and washed using a GeneChip Hybridization Wash and Stain Kit (Affymetrix), according to the manufacturer’s protocols. After being washed, the arrays were scanned using the GeneChip Scanner 3000 (Thermo Fisher Scientific). A human miRNA expression profiling chip (Shanghai Genechem Co. Ltd., Shanghai, China) was used for detection and data analysis. Genes with fold changes of >2 or ≤2 were analyzed using Affymetrix (GeneChip miRNA 4.0).

### 2.4. qRT-PCR analysis

The pre-osteoblast cell line MC3T3-E1 (ATCC) was cultured for 3 weeks in α-minimum essential medium (α-MEM; Invitrogen, Carlsbad, CA, USA) supplemented with 10 nM dexamethasone, 5 mM β-glycerophosphate, and 50 μg/mL ascorbic acid to induce osteoblastic mineralization [21]. Every 2 d, the medium (α-MEM) was replaced with fresh medium supplemented with an agomir (200 μM) and an antagomir (200 μM) of miR-30a-5p (GenePharma, Shanghai, China). The cells were harvested on day 21 and subjected to qRT-PCR to analyze of the expressions of the miRNAs identified in the extracted RNA samples. The sequences of PCR primers used are provided in Supplementary Table 1. The miRNA expression levels were quantified using a standard curve.

### 2.5. Luciferase assay

MC3T3-E1 cells (1 × 10^6^ cells/well) were maintained in 24-well plates. To analyze the 3′ untranslated region (UTRs) of the mRNAs of the target genes, we cotransfected MC3T3-E1 cells with wild-type or mutant luciferase reporter plasmids (Invitrogen, Carlsbad, CA, USA) and miR-30a-5p by using Lipofectamine 3000 (Invitrogen), according to the manufacturer’s instructions. After 24 h, we measured the luciferase activity on a dual luciferase-reporter assay system (Promega, Madison, WI, USA).

### 2.6. Alizarin red S and alkaline phosphatase staining

We analyzed the osteoblast-mediated generation of mineralized nodules in vitro by subjecting MC3T3-E1 cells to alizarin red S and ALP staining [22]. The cells were rinsed with phosphate-buffered saline (PBS) and fixed with 4% paraformaldehyde in PBS (pH, 7.4) for 15 min at room temperature. The cells were then washed 4 times with PBS, and stained with alizarin red S solution and ALP (Beyotime Biotechnology, Shanghai, China) at 4°C for 20 min. Finally, we calculated the percentage of the positively stained area per field of view.

The ALP activity was measured using an ALP assay kit (Beyotime Biotechnology, Shanghai, China), according to the manufacturer’s instructions.

### 2.7. Western blot analysis

We homogenized MC3T3-E1 cells in 200 µL lysis buffer, and incubated the lysed cells on ice for 30 min. The resultant protein fractions (3 μg/μL) were subjected to sodium dodecyl sulfate polyacrylamide gel electrophoresis, transferred to polyvinylidene difluoride membranes, and analyzed using western blotting. For this purpose, the membranes were incubated overnight with polyclonal antibodies against Runt-related transcription factor 1 (RUNX1) at 4°C, washed with Tris-buffered saline supplemented with Tween 20 at room temperature, and then, incubated again with the secondary antibodies. Quantification of the protein-band intensities was accomplished using densitometry (Gel Logic 2200; Rochester, NY, USA).

### 2.8. RUNX1 knockdown

To achieve stable RUNX1 knockdown, we infected MC3T3-E1 cells with a lentivirus-packaging short hairpin RNA (shRNA) expression vector (GenePharma, Shanghai, China) for 24–48 h, according to the manufacturer’s instructions. Chemically modified antisense oligonucleotides (antagomiR) have been used to inhibit miRNA expression *in vivo*. We used chemically modified miR-30a-5p antagomir (antagomiR-30a-5p) and miR-Ctrl antagomir (antagomiR-ctrl) purchased from GenePharma.

### 2.9. Ovariectomy protocol

To determine the effects of antagomiR-30a-5p on OP, we established an ovariectomy mouse model [23]. Female C57BL/6 mice were randomly assigned to the following groups (N = 8/group): (i) control group, in which the mice underwent a sham operation, (ii) an ovariectomy (OVX) group, in which mice were subjected to bilateral ovariectomy, and (iii) an OVX-antagomir group, in which ovariectomized mice were injected with antagomiR-30a-5p (100 nmol/kg) through the tail vein once every week. After 8 weeks, mice from all 3 groups were sacrificed, and their femurs were harvested for hematoxylin-eosin (H&E) staining. We observed decalcified bone slices under a fluorescence microscope. We measured the BMD, trabecular thickness (Tb.Th, mm), and trabecular separation (Tb.Sp, mm) of the distal femoral metaphysis on a high-resolution micro-computed tomography (CT) system (SkyScan-1176 micro-CT, Bruker, Belgium).

### 2.10. Statistical analysis

Values are expressed as the mean ± standard deviation of experiments performed in triplicate. Data were analyzed using SPSS version 20.0 (IBM, Armonk, NY, USA). Statistical analyses were carried out using the Pearson correlation test, Student *t* test, and one-way analysis of variance followed by a post hoc Tukey test. P values (two-tailed) less than 0.05 were considered to indicate significant differences.

## 3. Results

We observed that the level of miR-30a-5p was significantly increased in patients receiving long-term BP treatment for postmenopausal OP. The data demonstrated that miR-30a-5p was negatively correlated with bone formation. Consistent with this, *in vitro* osteoblast activity and matrix mineralization were increased by an antagomir of miR-30a-5p and decreased by an agomir of miR-30a-5p. We also found that miR-30a-5p directly targeted RUNX1 to inhibit osteoblastic differentiation. Moreover, miR-30a-5p antagomir administration promoted bone formation in ovariectomized mice.

### 3.1. Long-term BP treatment and differential miRNA expression

**Figure 1.**
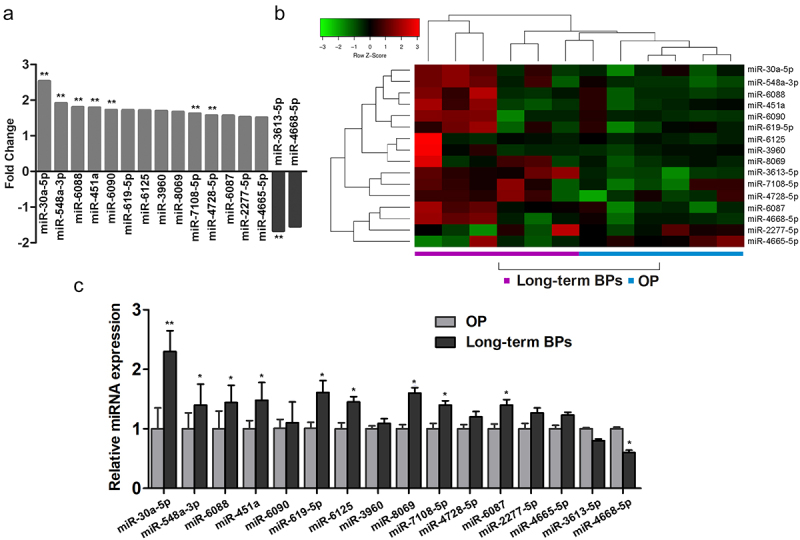
Expression of miR-30a-5p in serum samples obtained from women receiving long-term BP treatment for postmenopausal OP. **A**. Fold changes in the expressions of 14 differential miRNAs. **B**. A heat map prepared using hierarchical cluster analysis shows distinct miRNA expression patterns in the BP and control groups. **C**. Real-time PCR analysis of relative miRNA expression. *P < 0.05 and **P < 0.01 vs. the control group.

To identify differentially expressed miRNAs in OP patients undergoing long-term BP treatment, we assessed the miRNA expression profiles by using an Affymetrix Genechip microRNA 4.0 array. The detailed characteristics of the postmenopausal women in the long-term BP treatment and control groups are summarized in Supplementary Table 2. All the selected miRNAs were expressed in the serum samples obtained from the women in both groups. Compared to the miRNA levels in the control group, the BP group had 2 downregulated miRNAs (miR-3613-5p and miR-4668-5p) and 14 upregulated miRNAs (miR-30a-5p, miR-548a-3p, miR-6088, miR-451a, miR-6090, miR-619-5p, miR-6125, miR-3960, miR-8069, miR-7108-5p, miR-4728-5p, miR-6087, miR-2277-5p, and miR-4665-5p), all of which had fold changes of >1.5 (Figure 1a). A heat map of the miRNA expressions in both study groups is shown in Figure 1b.

We next verified the expression levels of the 16 differentially expressed miRNAs in serum specimens by using real-time PCR. Except for miR-4668-5p and miR-4665-5p, all the other miRNAs were expressed at remarkably higher levels in the serum specimens obtained from patients with postmenopausal OP undergoing long-term BP treatment than in the control serum specimens. We found that miR-30a-5p showed the most significant difference in expression (fold change, 2.4) between the BP and control groups (Figure 1c).

### 3.2. Correlation of miR-30a-5p expression with markers of bone formation and resorption

**Figure 2.**
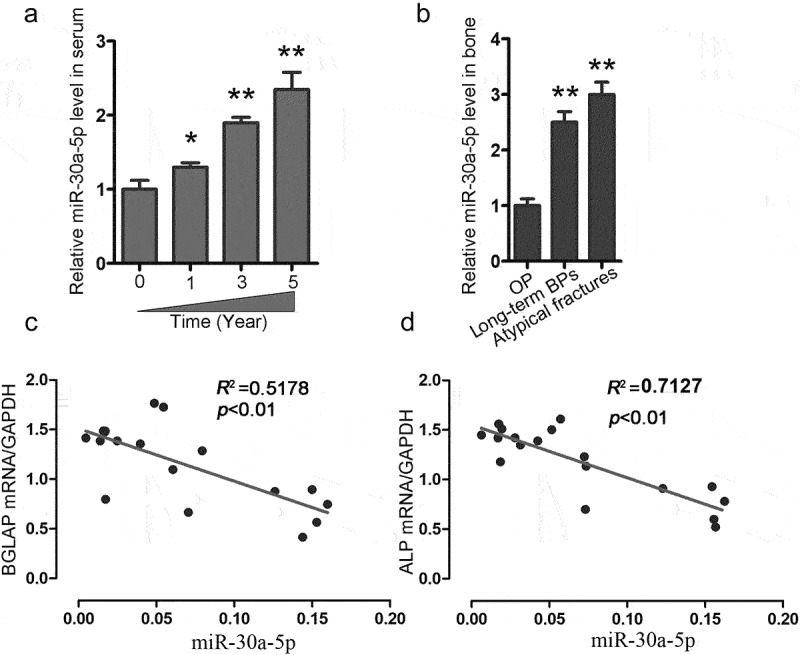
Increased miR-30a-5p expression is associated with reduced bone formation in OP patients receiving long-term BP treatment. **A**. qRT-PCR analysis of temporal changes in serum miR-30a-5p levels in OP patients undergoing BP treatment. *P < 0.05 and **P < 0.01 vs. the control group. **B**. qRT-PCR analysis of the changes in serum miR-30a-5p levels in patients with or without atypical fractures who were receiving BP treatment for postmenopausal OP. **P < 0.01 vs. the control group. **C**. Correlation of miR-30a-5p level and BGLAP mRNA level. **D**. Correlation of miR-30a-5p level and ALP mRNA level.

To clarify the significance of miR-30a-5p in the process of bone formation, we collected blood samples from patients receiving long-term BP treatment for postmenopausal OP. We found that long-term BP treatment was associated with increases in the serum expression of miR-30a-5p in postmenopausal OP patients (Figure 2a). Moreover, we observed that miR-30a-5p expression was significantly higher in postmenopausal OP patients with atypical fractures and in postmenopausal OP patients receiving long-term BP treatment than in the control subjects (Figure 2b). Furthermore, we found that the serum level of miR-30a-5p was negatively correlated with the mRNA levels of BGLAP and ALP, which are markers of bone formation (Figure 2c and 2d).

### 3.3. RUNX1 and miR-30a-5p

**Figure 3.**
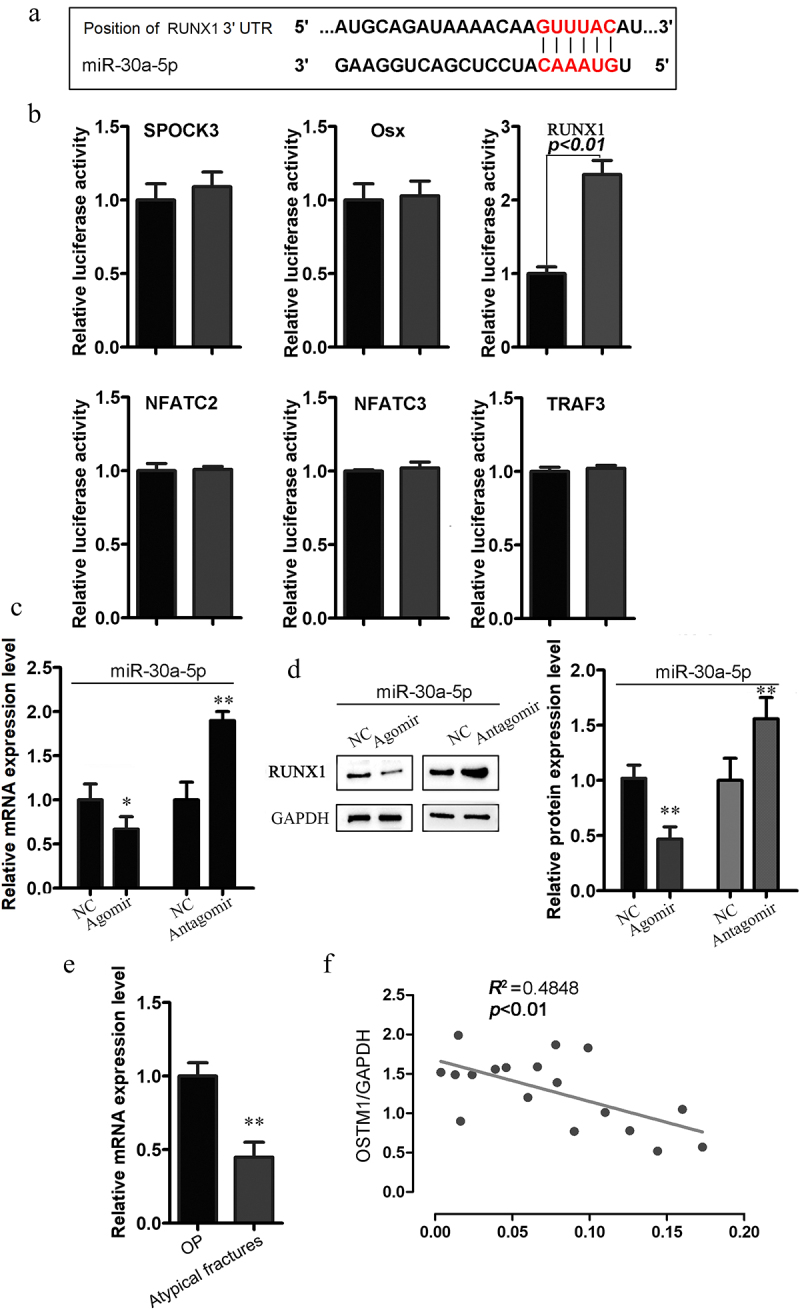
Relationship between miR-30a-5p and RUNX1. **A**. The predicted miR-30a-5p seed sequence in the RUNX1 3′UTR region. **B**. qRT-PCR analysis of the levels of the putative target genes of miR-30a-5p in osteoblasts treated with or without an miR-30a-5p agomir. **C**. RUNX1 mRNA level in osteoblasts treated with or without an miR-30a-5p agomir and an miR-30a-5p antagomir. **D**. RUNX1 protein level in osteoblasts treated with or without an miR-30a-5p agomir and an miR-30a-5p antagomir. **E**. Expression of RUNX1 in the bone tissues of postmenopausal OP patients with atypical fractures receiving long-term BP treatment, as compared with postmenopausal OP patients without atypical fractures. **F**. Correlation of miR-30a-5p level and RUNX1 mRNA level.

To investigate the molecular mechanisms underlying the miR-30a-5p-mediated inhibition of bone formation, we screened for putative miR-30a-5p targets by performing in silico complementarity searches of TargetScan, PicTar, and miRBase for key regulators of bone formation (Figure 3a). From the predicted genes, we selected 6 putative miR-30a-5p target genes, all of which are transcription factors or signaling molecules involved in bone formation (Figure 3b). We assessed the fold changes in the luciferase readout from the 3ʹ-UTR reporter for each candidate target gene after cotransfection with MC3T3-E1 cells with or without an miR-30a-5p agomir (Figure 3a). We found that the miR-30a-5p agomir markedly inhibited luciferase activity in cells transfected with reporter plasmids containing RUNX1, as compared with control cells, indicating that this gene could be a legitimate target of miR-30a-5p. Consistent with these findings, real-time PCR and western blotting showed that RUNX1 expression was upregulated by an miR-30a-5p antagomir and downregulated by an miR-30a-5p mimic (Figure 3c and 3d). Moreover, among postmenopausal OP patients receiving long-term BP treatment, RUNX1 expression in bone tissues was lower among those with atypical fractures than among those without atypical fractures (Figure 3e). We also observed a negative correlation between the miR-30a-5p level and RUNX1 expression in the serum specimens obtained from patients receiving long-term BP treatment for postmenopausal OP (Figure 3f). These results indicated that miR-30a-5p negatively regulated RUNX1.

### 3.4. Inhibition of osteoblast activity by miR-30a-5p

**Figure 4.**
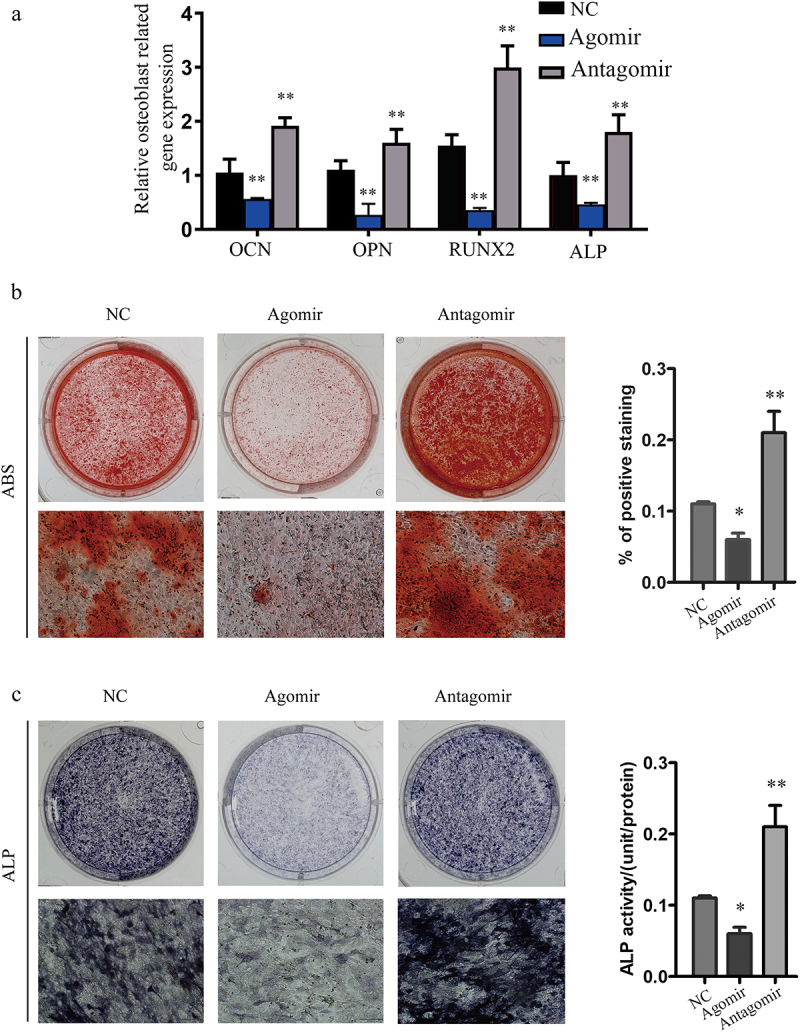
Inhibition of osteoblast activity by miR-30a-5p. **A**. RT-PCR analysis of genes related to the osteoblastic differentiation of MC3T3-E1 cells treated with or without an miR-30a-5p agomir/antagomir. *P < 0.05 and **P < 0.01. The error bars show the standard error of the mean. **B**. Effect of miR-30a-5p on the generation of alizarin red S-positive cells. MC3T3-E1 cells were cultured for 21 days and analyzed for the amount of alizarin red S staining. *P < 0.05 and **P < 0.01. **C**. Effect of miR-30a-5p on ALP staining and ALP activity. *P < 0.05 and **P < 0.01.

To measure the osteoblast activity of miR-30a-5p agomir or antagomir on the precursors of osteoblast, we transfected MC3T3-E1 cells with miR-30a-5p agomir or antagomir. Significant downregulation of Runt-related transcription factor 2 (RUNX2), ALP, osteocalcin (OCN), and osteopontin (OPN) expression was observed in cells transfected with the miR-30a-5p agomir, while in cells transfected with the miR-30a-5p antagomir, the expression of these osteoblast-related genes was obviously increased at the mRNA level (Figure 4a).

To evaluate the effect of miR-30a-5p on osteoblast differentiation, we measured calcium salt deposition and ALP activity in MC3T3-E1 cells transfected with the miR-30a-5p agomir or antagomir. Alizarin red S staining revealed that matrix mineralization of the transfected cells was decreased in response to the miR-30a-5p agomir and increased in response to the miR-30a-5p antagomir (Figure 4b). Finally, ALP staining and ALP activity was suppressed in response to the miR-30a-5p agomir and enhanced in response to the miR-30a-5p antagomir (Figure 4c).

### 3.5. RUNX1 is crucial for the miR-30a-5p-mediated inhibition of osteoblastic differentiation

**Figure 5.**
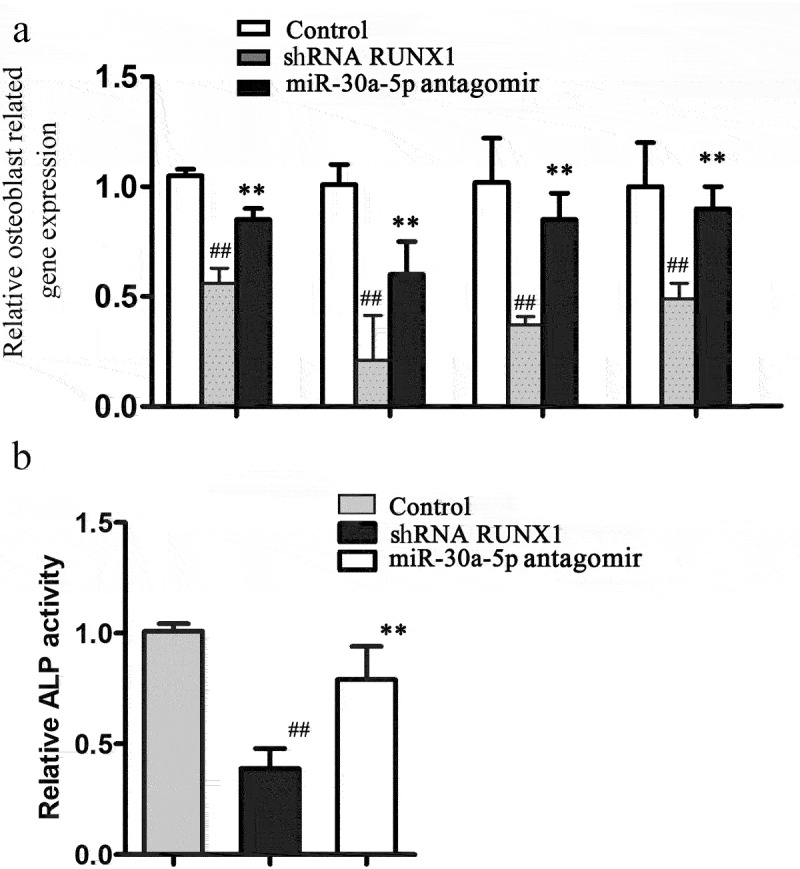
RUNX1 knockdown reverses the effects of miR-30a-5p antagomir on osteoblastic differentiation. **A**. RT-PCR analysis of genes associated with the osteoblastic differentiation of MC3T3-E1 cells treated with or without an miR-30a-5p agomir. **B**. Effect of miR-30a-5p on ALP activity. ^##^P < 0.01 compared with the control group. **P < 0.01 compared with the shRNA group.

To determine if miR-30a-5p inhibited osteoblastic differentiation by targeting RUNX1, we used shRNA to knock down RUNX1 expression in MC3T3-E1 cells. RUNX1 protein expression was determined using western blotting (Figure S2). We found that qRT-PCR analysis indicated that in MC3T3-E1 cells, RUNX1 knockdown significantly suppressed osteoblastic differentiation by suppressing OCN, OPN, RUNX2, bone sialoprotein (BSP), and ALP expression. The miR-30a-5p antagomir reversed the RUNX1 knockdown-mediated decreases in OCN, OPN, RUNX2, BSP, and ALP expression in MC3T3-E1 cells. Consistent with these findings, ALP activity was deceased by RUNX1 knockdown, and this decrease was reversed by the miR-30a-5p antagomir (Figure 5a and 5b). These findings indicated that miR-30a-5p suppressed osteoblastic differentiation by targeting RUNX1.

### 3.6. In vivo *inhibition of bone formation by miR-30a-5p*

**Figure 6.**
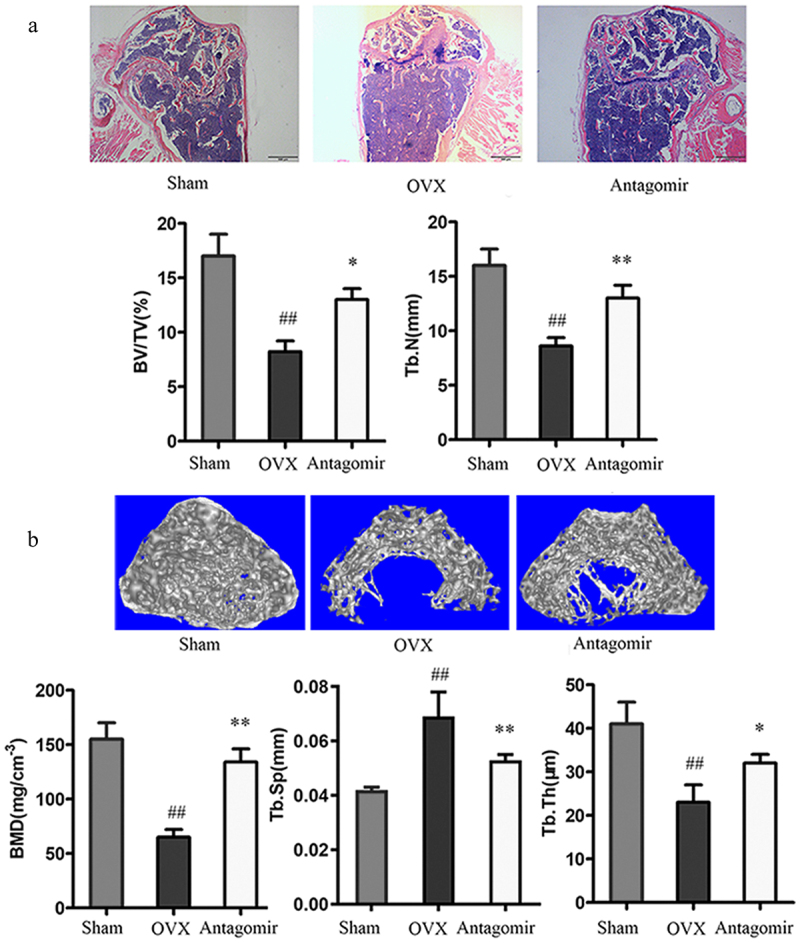
Effect of miR-30a-5p antagomir on *in vivo* bone formation. OVX mice were administered miR-30a-5p antagomir for 4 weeks. **A**. H&E staining of bone tissue (scale bars, 500 μm). Quantitative analyses of histomorphometric bone parameters of trabecular bone volume per total volume (BV/TV) and the trabecular number (N. trabecular) per square millimeter was evaluated. ##P < 0.01 compared with the sham group. *P < 0.05; **P < 0.01 compared with the OVX group. N = 8 for each group. **B**. Micro-CT reconstruction of the distal femur metaphysis in the OVX mice and miR-30a-5p antagomir-treated mice (scale bars, 0.2 mm). Micro-CT was also used to measure BMD, Tb.Sp, and Tb.Th at the distal femur metaphysis in the OVX mice and miR-30a-5p antagomir-treated mice. ^#^P < 0.05; ^##^P < 0.01 compared with the sham group. *P < 0.05; **P < 0.01 compared with the OVX group. N = 8 for each group.

Since we found that miR-30a-5p regulated osteoblastic differentiation and mineralization *in vitro*, we attempted to determine if this miRNA also reduced bone formation *in vivo*. Therefore, we prepared an OP mouse model by using ovariectomy. Mice which had undergone a sham operation or ovariectomy were administered miR-30a-5p antagomir via tail vein injection. In order to observe the microstructure changes of cancellous bone, H&E staining were conducted. In the OVX mice, there was a decrease in the number and trabecular bone volume in the distal femur, as compared to the sham-operated controls. Treatment of the OVX mice with the miR-30a-5p antagomir dramatically increased in the number and trabecular bone volume (Figure 6b). Micro-CT demonstrated that miR-30a-5p antagomir treatment attenuated the OVX-induced deteriorations observed in the distal femur, namely, poor organization of the trabecular architecture and low bone mass (Figure 6c). Micro-CT also revealed that the bone histomorphometric parameters (BMD, BV/TV, and Tb.Th) were significantly higher in the OVX-antagomir group than in the OVX group. These findings suggested that the inhibition of miR-30a-5p increased bone formation by regulating osteoblastic differentiation.

## 4. Discussion

BPs are widely prescribed and highly effective at limiting bone loss in OP. Although they are generally very well tolerated, potential adverse effects may limit BP use in some patients. In this study, we investigated novel indicators for the early prediction of adverse reactions during the long-term use of BPs, and described the potential underlying molecular mechanism. The present study showed that miR-30a-5p expression is affected by long-term BP treatment for postmenopausal OP. The serum miR-30a-5p level was found to be negatively correlated with markers of bone formation. In addition, miR-30a-5p inhibited osteoblast activity *in vitro* by targeting RUNX1 expression and suppressed *in vivo* bone formation in an OVX mouse model.

Currently, BPs are the most widely used drugs for the treatment of OP. Indeed, BPs are listed as the first-line treatment for postmenopausal OP and male OP in the clinical guidelines for the prevention and treatment of osteoporosis issued by the National Osteoporosis Guideline Group, as these drugs have well-documented anti-fracture efficacy in OP. However, prolonged BP treatment is associated with the incidence rates of atypical femoral fractures and jaw osteonecrosis; therefore, after 5 years of oral BP treatment or 3 years of intravenous BP treatment, a reassessment of the risk of adverse events should be conducted [2]. In this study, using gene chip technology, we found that elevated serum levels of miR-30a-5p were closely linked with suppression of bone formation, as indicated by the negative correlation of miR-30a-5p expression with BGLAP and ALP mRNA levels in OP patients receiving long-term BP treatment. We also found that among postmenopausal OP patients receiving prolonged BP treatment, the miR-30a-5p level was higher in those with atypical fractures than in those without fractures. MiRNAs contribute to the regulation of many biological processes, and their abnormal expression/function has been linked to the incidence, development, and prognosis of diseases such as OP [24]. As biomarkers of disease and potential therapeutic targets, miRNAs have rapidly garnered research and clinical attention [25]. Our results indicate among patients receiving prolonged BP treatment for postmenopausal OP, the miR-30a-5p level is abnormally elevated, and that this may result in the inhibition of osteoblast-mediated bone formation.

It has been reported that miRNAs are crucially involved in several pathophysiological processes, such as bone remodeling [26]. However, the involvement of and molecular mechanisms underlying the alterations in the expression of miRNAs and their target genes involved in bone regulation under the influence of BP-induced bone turnover remain poorly understood. This study found that miR-30a-5p directly inhibits osteoblastic differentiation in MC3T3-E1 cells. Osteoclasts are the primary bone-resorbing cells in the human body, and are responsible for postmenopausal OP. A previous study reported that miR-30a-5p failed to inhibit osteoclastogenesis in bone marrow macrophages stimulated using macrophage colony-stimulating factor and RANKL (receptor activator of nuclear factor kappa-Β ligand). This may be because miR-30a-5p regulates bone metabolism via an antagomir of osteoblast differentiation.

RUNX1 is a key transcription factor that belongs to the RUNX1 family of genes, which is a major signaling pathway regulating hematopoietic stem cells and hematopoiesis [27]. A study has shown that BMP9 promotes the endogenous expression of RUNX1 in murine MSC lines and murine multi-lineage cell lines [28]. The overexpression of RUNX1 resulted in an increase in osteogenic differentiation, whereas knockdown of RUNX1 inhibited osteogenic differentiation in MSC lines and murine multi-lineage cell lines [21]. These results suggested that RUNX1 may be an essential modulator of the osteogenic differentiation of MSCs. In the current study, we found that RUNX1 can be directly targeted by miR-30a-5p, and might be the mechanism via which miR-30a-5p inhibits osteoblastic differentiation.

Although this study represents the first integrated analysis of miRNA expression in patients receiving long-term BP treatment, some limitations should be noted. First, miRNA expression profiles were compared between patients receiving long-term oral BP treatment and patients not receiving oral BPs. Future experiments will compare miRNA expression profiles between patients receiving long-term BP treatment and those receiving other anti-osteoporotic drugs, which are also likely to affect miRNA expression. Second, the expression and diagnostic potential of miR-30a-5p were studied using serum samples; the diagnostic value of miR-30a-5p during long-term BP treatment for postmenopausal OP requires further investigation using tissue samples. Third, although we evaluated the clinical significance of miR-30a-5p, its biological functions and relationships in long-term BP treatment for postmenopausal OP require further investigation using both *in vivo* and *in vitro* experiments.

## 5. Conclusion

In summary, our results demonstrate that miR-30a-5p expression is affected by long-term treatment with BPs in patients with postmenopausal OP, as compared to control subjects. Furthermore, our results showed that miR-30a-5p inhibited osteoblast activity by targeting RUNX1. The increase in the serum expression of miR-30a-5p in women receiving long-term BP treatment for postmenopausal OP showed a significant negative correlation with bone formation. Consistent with this, treatment with an antagomir of miR-30a-5p increased bone formation *in vivo*. Thus, our study reveals that miR-30a-5p is an antagomir of osteoblastic bone formation in patients receiving long-term BP treatment for postmenopausal OP. We identified miR-30a-5p as a novel biomarker and clinical indicator for monitoring OP in patients receiving long-term BP treatment. Furthermore, miR-30a-5p is a candidate therapeutic target in OP.

## Supplementary Material

Supplemental Material
