# Supplementary material for: Suppression of bone remodeling associated with long-term bisphosphonate treatment is mediated by microRNA-30a-5p
Source: Bioengineered. 2022 Apr 12;13(4):9741–53. doi: 10.1080/21655979.2022.2060584 (PMC9161941; doi:10.1080/21655979.2022.2060584)

**Supplementary Table 1. Primers used in QRT- PCR**

| **Gene** | **RT primer article number** | **Upstream primer article number** | **Downstream primer article number** |
| --- | --- | --- | --- |
| U6 | SSD904071008 | SSD0904071006 | SSD0904071007 |
| hsa-miR-30a-5p | SSD809230210 | SSD809230902 | SSD089261711 |
| BGLAP | SSD1392710131 | SSD1392710132 | SSD089261711 |
| ALP | SSD809230378 | SSD809231070 | SSD089261711 |
| [RUNX1](http://www.ensembl.org/Homo_sapiens/Gene/Summary?g=ENSG00000124813.16) | SSD809230485 | SSD809231177 | SSD089261711 |
| RUNX2 | MIR8001466 | MIR8001467 | SSD089261711 |
| OCN | MIR8001378 | MIR8001379 | SSD089261711 |
| OPN | MIR8000569 | MIR8000570 | SSD089261711 |

**Supplementary Table 2 Clinical features of Patients**

| **Patient** | **Age** | **T score for BMD at Lumbar Spine** | **Persistence with oral BPs(years)** |
| --- | --- | --- | --- |
| 1 | 65 | -1.55 | 5 |
| 2 | 63 | -1.43 | 2 |
| 3 | 77 | -1.29 | 3 |
| 4 | 78 | -1.39 | 2 |
| 5 | 79 | -1.79 | 1 |
| 6 | 80 | -1.89 | 2.5 |
| 7 | 79 | -1.98 | 3.5 |
| 8 | 85 | -2.10 | 2.5 |
| 9 | 58 | -1.87 | 2.6 |
| 10 | 65 | -1.67 | 5 |
| 11 | 59 | -1.34 | 2 |
| 12 | 77 | -1.56 | 3 |
| 13 | 74 | -1.58 | 4 |
| 14 | 73 | -1.67 | 3 |
| 15 | 72 | -1.88 | 3 |
| 16 | 79 | -2.03 | 4 |
| 17 | 80 | -2.07 | 5 |
| 18 | 79 | -1.90 | 3 |
| 19 | 81 | -1.65 | 3.5 |
| 20 | 82 | -1.54 | 3.5 |
| 21 | 85 | -1.32 | 4 |
| 22 | 84 | -1.26 | 3.5 |
| 23 | 83 | -1.78 | 2 |
| 24 | 81 | -1.98 | 2.5 |
| 25 | 80 | -1.87 | 3 |
| 26 | 79 | -1.91 | 2.5 |
| 27 | 80 | -2.02 | 3 |
| 28 | 79 | -2.12 | 5 |
| 29 | 77 | -1.79 | 2 |
| 30 | 72 | -1.59 | 3 |

**Supplementary Figure1**

Ethical approval


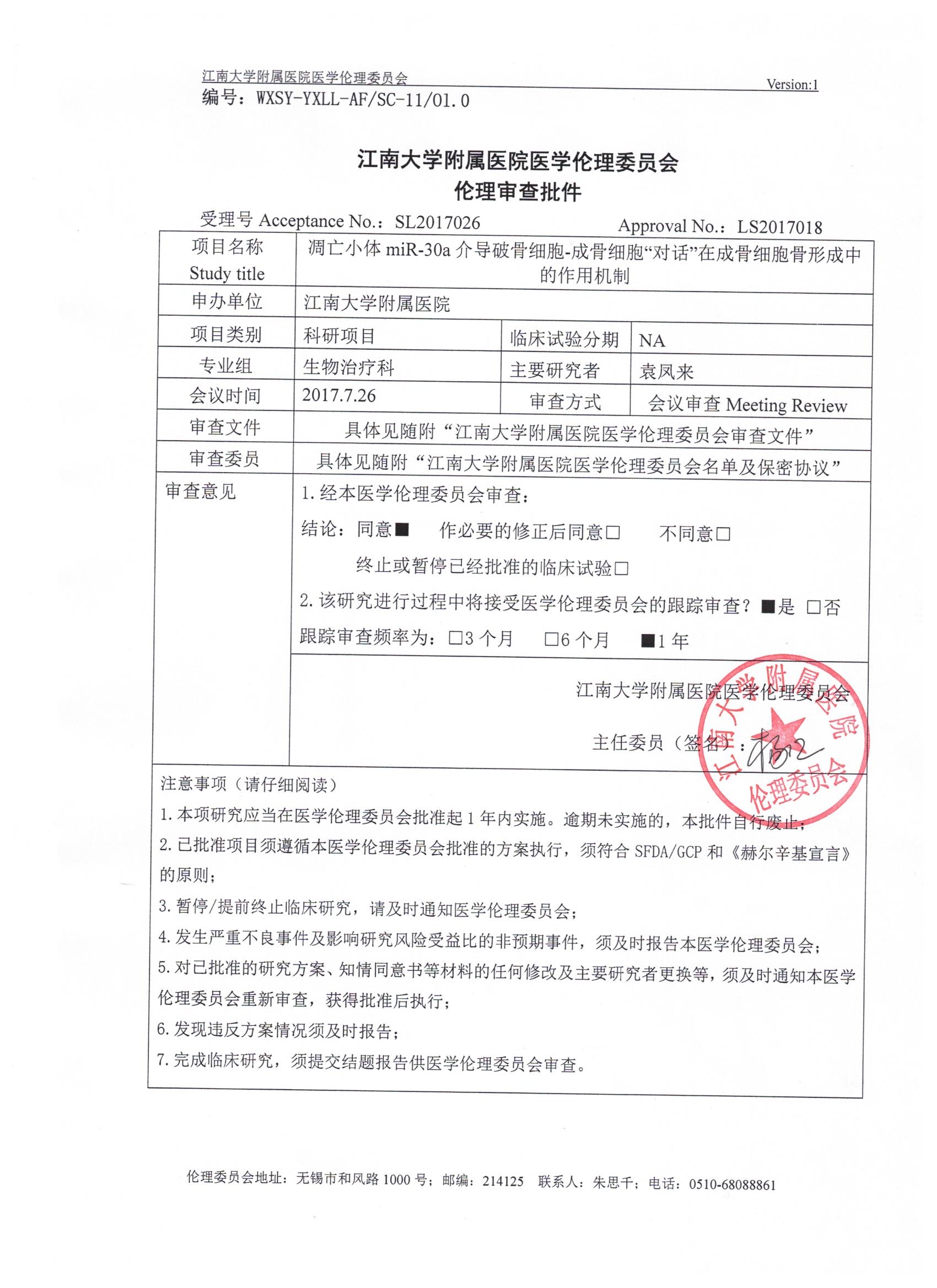


**Supplementary Figure2**

western blot images


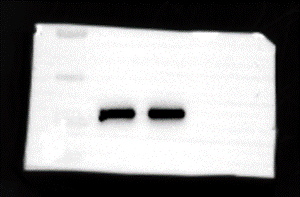

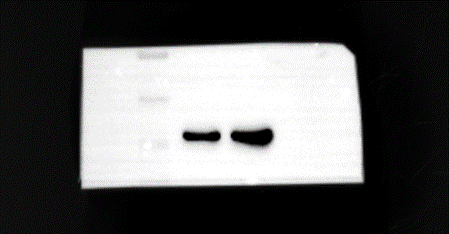

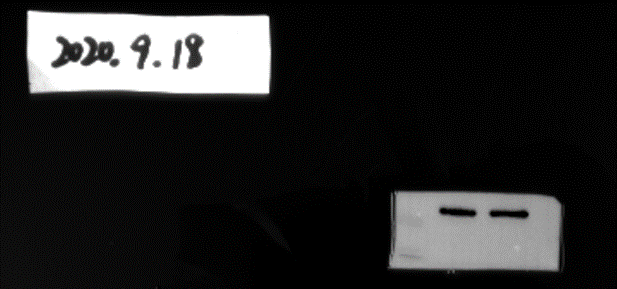

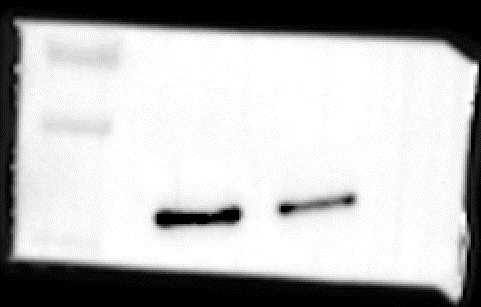

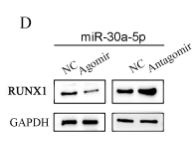

Supplement: Supplemental Material [file KBIE_A_2060584_SM1498.docx]
